# Impact of sunflower seed oil versus mustard seed oil on skin barrier function in newborns: a community-based, cluster-randomized trial

**DOI:** 10.1186/s12887-019-1871-2

**Published:** 2019-12-23

**Authors:** Aimee Summers, Marty O. Visscher, Subarna K. Khatry, Jeevan B. Sherchand, Steven C. LeClerq, Joanne Katz, James M. Tielsch, Luke C. Mullany

**Affiliations:** 1 Department of International Health, Johns Hopkins Bloomberg School of Public Health, 615 N. Wolfe Street, W5009, Baltimore, MD 21205 USA; 2 Skin Sciences Program, Cincinnati Children’s Hospital Medical Center, 3333 Burnet Avenue, Cincinnati, OH 45229 USA; 3 Nepal Nutrition Intervention Project-Sarlahi (NNIPS), Kathmandu, Nepal; 4 Department of Microbiology, Institute of Medicine, Tribhuvan University Teaching Hospital, Maharajgunj Rd, Kathmandu, 44600 Nepal; 5 Department of Global Health, Milken Institute School of Public Health, George Washington University, 950 New Hampshire Avenue, Washington, DC USA

**Keywords:** Skin, Skin barrier, Stratum corneum, Neonate, Emollient therapy, Mustard oil, Sunflower oil, Massage, TEWL, pH, Skin condition

## Abstract

**Background:**

Natural vegetable oils are widely used for newborn massage in many low resource settings. Animal models indicated that sunflower seed oil (SSO) can accelerate skin barrier recovery following damage, while other oils, including mustard oil (MO), may cause further skin barrier damage. The objective was to compare the effects of two SSO and MO used for routine massage on skin integrity in premature and full-term neonates.

**Methods:**

This community-based cluster randomized controlled trial included 995 neonates assigned to full body massage with sunflower seed oil (SSO, intervention) or mustard seed oil (MO, standard practice) from July 2012–May 2014 in Sarlahi, Nepal. Skin integrity measures were evaluated over 28 days, including skin condition (erythema, rash, dryness), skin surface pH, stratum corneum (SC) cohesion/protein concentration, and transepidermal water loss (TEWL). Overall means and rates of change in these skin measures were compared between oil groups using bivariate random-effects models.

**Results:**

500 and 495 live born neonates received repeated massage with MO and SSO, respectively. Skin pH decreased more quickly for SSO than MO in the first week of life, with a difference in mean daily reductions of 0.02 (95% CI: 0.002–0.040). Erythema, rash and dryness increased (worsened) over days 1–14 then decreased by day 28, with no significant oil group differences. TEWL increased over time, with no significant oil group differences. Gestational age did not modify the effect; the slightly faster decrease in skin pH among SSO infants was similar in magnitude between term and preterm infants.

**Conclusions:**

Oil type may contribute to differences in skin integrity when neonates are massaged regularly. The more rapid acid mantle development observed for SSO may be protective for neonates in lower resource settings.

**Trial registration:**

ClinicalTrials.gov (NCT01177111); registered August 6th, 2010.

## Background

Neonatal stratum corneum (SC) and epidermis provide robust innate immunity in addition to antimicrobial defense, protection from ultraviolet radiation, water loss, injury, and thermoregulation [1, 2]. The premature infant skin barrier is underdeveloped, thereby creating risks for increased permeability, skin damage, delayed barrier maturation, and infection [3, 4]. In low resource settings, where the majority of neonatal deaths occur [5, 6], skin care based interventions have emerged as important strategies in improving survival, especially among low birth weight babies and those born preterm. In particular, given widespread use of natural vegetable oils for newborn massage in many low resource settings, interest in emollient therapy remains high. Animal models have shown that sunflower seed oil (SSO) can accelerate skin barrier recovery following damage, while other oils, including mustard oil (MO), cause further barrier damage [7]. The lipid profile of SSO may enhance skin barrier integrity and function by providing 1) a physical barrier to infectious agents, 2) essential SC fatty acids and 3) linoleic acid that may reduce epidermal inflammation [7]. Hospital-based studies have demonstrated improved skin condition scores, reductions in nosocomial infections, and improved survival among preterm infants receiving applications of SSO [8, 9]. Such results provided strong rationale for evaluating this intervention in a community setting, such as Nepal, where almost half of deliveries occur at home [10]. Newborns in Nepal are routinely massaged with MO several times daily throughout the neonatal period and beyond [11]. The vigorous, lengthy massages might increase transcutaneous acquisition of invasive environmental pathogens, particularly among preterm infants whose skin barrier is underdeveloped and fragile [12]. Compromised barrier function occurs with nutritional deficiencies that are common to the region [13]. We previously described skin barrier function and integrity indicators among preterm and full term infants massaged routinely with MO in rural communities of Sarlahi District, Nepal [14]. We observed that skin rash and erythema increased (worsened) until day 14 then decreased by day 28. Dynamics of skin pH reduction over 28 days for all infants reflected skin barrier maturation, but this process was delayed among preterm infants. Here we quantify differences in skin barrier integrity and maturation between newborns randomly assigned to routine massage with MO or SSO over the first postnatal month.

## Methods

### Setting and parent trial

Our cluster-randomized study was nested within a larger trial (ClinicalTrials.gov, NCT01177111) of the impact of topical skin emollient application on newborn mortality and morbidity in Sarlahi District, Nepal. In that parent trial, field workers identified incident pregnancies through universal five-weekly home visits, enrolled pregnant women, and followed them through delivery and the first month post-partum. Visits at enrollment and during monthly pregnancy visits allowed for collection of household socioeconomic status, paternal and maternal demographic variables, prior birth history, and care-seeking and morbidity during pregnancy. Post-delivery home visits started as soon as possible (i.e. day 1), and continued on days 3, 7, 10, 14, 21, and 28. On day 1, data collectors recorded the circumstances of labor, delivery, and immediate postpartum care, inquired about infant health since delivery, and measured weight (BD-585 Pediatric Scale [10 g precision], Tanita Corporation of America, Arlington Heights, IL). At subsequent visits workers collected maternal reports of infant morbidities and examined babies for signs of infection.

### Intervention and randomization

Communities in the larger trial were randomized to either promotion of full-body topical applications (“newborn massage”) with SSO (intervention) or the MO (comparison), traditionally practiced in Nepal almost universally [11]. The study area encompassed 34 village development committees (VDC, a government defined unit), further divided by our team into “clusters” (unit of randomization) based on population. For clusters with prior neonatal death data, restricted randomization was performed to ensure balance on mortality risk. The remaining clusters were randomized with a computerized quasi-random number generator by author LCM prior to trial initiation. Blinding of field workers and mothers was not possible given the distinct colors and smells of MO and SSO. Both oils were purchased (Shiv Shakti Ghee Udyhog Pvt. Ltd., Jitpur, Nepal) approximately every 4–6 weeks and stored in sealed half-liter plastic packets at room temperature at site headquarters. Samples were submitted to the Government of Nepal Food Inspection Laboratory (Hetauda, Makwanpur District, Nepal) for quality assurance. In late pregnancy (~ 28–32 weeks), field workers visited enrolled women to promote the use of either SSO or MO and provide a 100 ml bottle of oil with instruction to initiate full body massage using the provided oil as soon as possible after birth. During week one, field workers visited daily to promote oil use and resupply households with 500 ml bottles on days 1, 10, and 21.

### Design of Current Study

Our study was nested within the larger trial, as previously described [14]. A subset of newborns based on geographic area, gestational age (GA) and timing of the first postnatal visit were enrolled on day 1. We chose a subset of 9 VDCs close to our main field office to maintain a cold chain for specimens. All preterm and a randomly selected 20% sample of term infants born alive between July 2012 and May 2014, and met by field workers before age 48 h were eligible. This down-sampling of term infants was to achieve a ratio of term/preterm infants closer to unity, increasing power to explore if GA modified the relationship between oil choice and skin integrity.

### Measurement methods

After obtaining informed consent from mothers for infant participation, a specialized field worker team collected data on days 1, 3, 7, 14, and 28 [14]. They used validated scoring scales for erythema, rash and dryness to assess skin condition at the chest, right arm, and left leg (Additional file 1: Table S1) [15, 16]. After four training days, field workers’ scores from photographs were compared to an expert (author MOV) with a kappa of 0.88. During implementation, their scores were periodically compared to an investigator (author AS) with an agreement of 95%. Transepidermal water loss (TEWL, g/m^2^/h), relative humidity and temperature were measured using a closed chamber device (VapoMeter, Delfin Technologies, Kuopio, Finland) [17]. Three chest skin pH measurements (Skincheck, Hanna Instruments, Bedfordshire, UK) were averaged. Chest SC cohesion was determined as protein concentration from 380 mm^2^ D-Squame discs (CuDerm, Dallas, TX, USA). Protein concentration was quantified as optical density (SquameScan 850A (Heiland electronic, Wetzlar, Germany) [18].

### Statistical analysis

We aimed to enroll 1000 infants equally stratified by preterm (< 37 weeks) and full term to detect differences in skin scores, skin pH, TEWL, and protein concentrations of 0.5 with 71–100% power for standard deviations of 1.0–2.5. Analyses were conducted using Stata v15 (College Station, TX). Biologically implausible outliers (TEWL > 100 g/m^2^/hr. and skin pH > 8 and < 3) were removed as being due to a confounding biological process (e.g. perspiration rather than TEWL) [14].

Maternal, paternal, and newborn characteristics (sex, GA, birthweight, small for gestational age status, time to breastfeeding initiation, time between the first visit and the most recent massage, and the number of massages per day during the first week) were examined by group to assess randomization balance. Visit-specific ambient temperature and relative humidity were converted to heat index [19, 20]. Means and standard deviations of all skin measures per visit were compared between oil groups using bivariate random-effects models, accounting for clustering and repeated measures. Locally weighted scatterplot smoothing curves were created to show the changes of skin barrier integrity measures over time for each oil group. We conducted sub-group analyses stratified by GA (full-term ≥37 weeks, late preterm 34–36 weeks, early preterm < 34 weeks). Analyses followed an intention-to-treat approach for participating infants, regardless of the actual allocation to, provision of, or compliance with study-provided oils. Infants who missed visits were not included in the analyses for that visit, but were included in analyses if met on future visits.

### Ethical approval

The Ethical Review Committee of the Institute of Medicine, Tribhuvan University (Kathmandu, Nepal) and the Institutional Review Board of the Johns Hopkins Bloomberg School of Public Health (Baltimore, MD) approved the study. Mothers provided verbal consent for their infant’s participation.

## Results

**Fig. 1:**
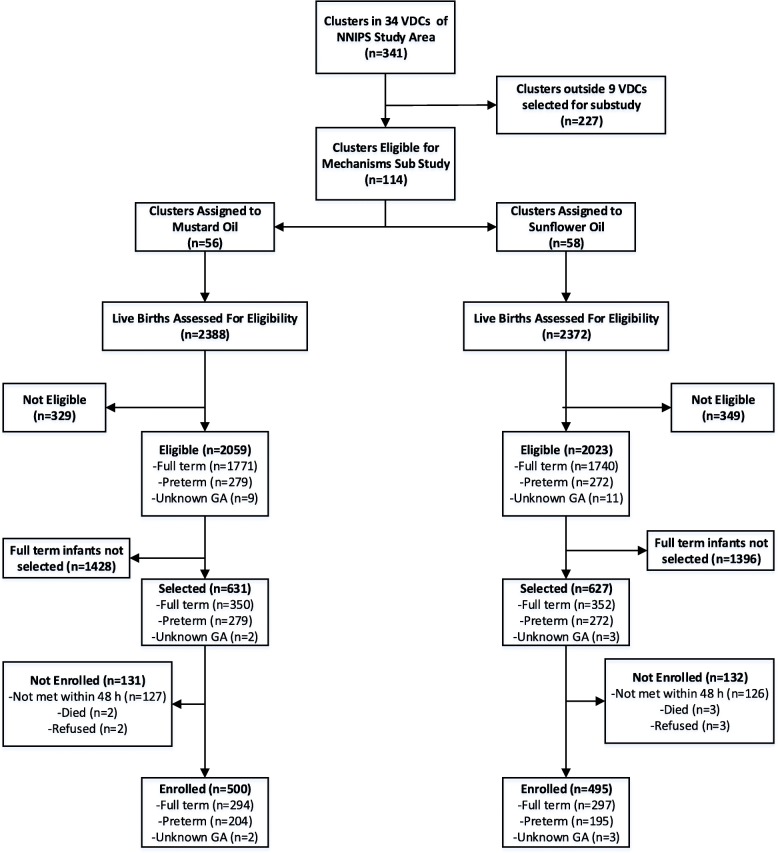
Participant Flowsheet. For SSO and MO, 11 (2.2%) and 10 (2.0%) infants died and 67 (13.5%) and 74 (14.8%) moved out of the study area prior to age 28 days, respectively

Between July 23rd 2012 and May 18th, 2014, 702 (a 20% random sub-sample of 3511) full term and 551 preterm live born infants were potentially eligible (Fig. 1). Of these, 253 (20.2%) were not visited before age 48 h and 5 (0.4%) died prior to the field worker’s arrival. Among the 1000 thus recruited, 5 (0.4%) mothers declined their infant’s participation, leaving 995 participating infants (500 and 495 in the MO and SSO groups, respectively). Three hundred ninety-four (78.8%) and 395 (79.8%) infants (MO and SSO groups, respectively) completed the 28-day follow-up. For SSO and MO, 11 (2.2%) and 10 (2.0%) infants died and 67 (13.5%) and 74 (14.8%) moved out of the study area prior to age 28 days, respectively.

Household, maternal and newborn characteristics were well-balanced (Table 1) between oil groups, with similar proportions of males and females, preterm and term babies, low birthweight, and small-for-gestational age (SGA) in each group. The prevalence of low birthweight and SGA infants was 36.4 and 45.6%, respectively, and comparable to the general Nepali population [21]. The proportion of preterm infants was slightly higher for MO (40.9%) versus SSO (39.7%). Massage frequencies during week one were similar (4.6 and 4.2 per day for MO and SSO, respectively). Compliance with oil application and massage was 100%. Compliance was measured if the infant had been massaged with the correct oil for the intervention group they were assigned. Relative humidity was over 80% (mean 75.1% ± 13.2%, range 10% (*n* = 3) to 100% (*n* = 5)) for 45.6% of 2301 home visits. Mean temperature and heat index were 30.0 °C (±4.5) and 33.6 °C (±9.0), respectively. There were no differences in skin infections by oil group.

**Table 1:** Baseline household, maternal, and newborn characteristics of neonates by oil groups, Sarlahi, Nepal

	Oil Randomization Group			
	Mustard Oil		Sunflower Oil	
	*n*	(%)	*n*	(%)
Ethnic Group	*N* = 500		*N* = 494	
Pahadi	23	4.6	37	7.5
Madeshi	477	95.4	457	92.5
Maternal literacy	*N* = 500		*N* = 494	
Literate	147	29.4	132	26.7
Paternal literacy	*N* = 500		*N* = 494	
Literate	278	55.6	275	55.7
Maternal Education	*N* = 500		*N* = 495	
None	351	70.2	361	72.9
1–5 yrs	48	9.6	41	8.3
6–10 yrs	79	15.8	71	14.3
> 10 yrs	22	4.4	22	4.4
Paternal Education	*N* = 500		*N* = 495	
None	219	43.8	216	43.6
1–5 yrs	77	15.4	76	15.4
6–10 yrs	165	33.0	169	34.1
> 10 yrs	39	7.8	34	6.9
Maternal Age	*N* = 500		*N* = 494	
< 18 yrs	55	11.0	52	10.5
18–24 yrs	295	59.0	300	60.7
25–29 yrs	105	21.0	100	20.2
> =30 yrs	45	9.0	42	8.5
Parity	*N* = 500		*N* = 494	
None	145	29.0	136	27.5
1–2	219	43.8	229	46.3
3–4	114	22.8	113	22.8
> 5	22	4.4	17	3.4
Antenatal Care Visits	*N* = 500		*N* = 494	
No ANC	142	28.4	146	29.6
1–2 ANC visits	188	37.6	182	36.8
3–4 ANC visits	148	29.6	146	29.6
> =5 ANC visits	22	4.4	20	4.0
Location of Delivery	*N* = 500		*N* = 494	
Home	360	72.0	339	68.6
Facility	140	28.0	155	31.4
Sex	*N* = 500		*N* = 494	
Male	269	53.8	260	52.6
Female	231	46.2	234	47.4
Gestational Age	*N* = 498		*N* = 491	
< 34 wks	56	11.2	56	11.4
34–36 wks	148	29.7	139	28.3
≥ 37 wks	294	59.1	296	60.3
Birthweight	*N* = 498		*N* = 493	
< 1500 g	6	1.2	13	2.6
1500-2499 g	161	32.3	183	37.1
≥ 2500 g	331	66.5	297	60.3
SGA^1^ Status	*N* = 498		*N* = 493	
AGA^2^	268	53.8	269	54.6
SGA 3–10%	110	22.1	95	19.3
SGA 3%	120	24.1	129	26.2
Time to breastfeeding initiation (hrs)	*N* = 411		*N* = 427	
< 1	138	33.6	140	32.8
1–2	225	54.7	227	53.2
3–4	29	7.1	30	7.0
≥ 5	19	4.6	30	7.0
Time between first visit and last massage (minutes)	*N* = 496		*N* = 491	
< 30	117	23.6	96	19.6
30–59	84	16.9	85	17.3
60–119	128	25.8	136	27.7
120–179	62	12.5	70	14.3
≥ 180	105	21.2	104	21.2
	Mean	SD	Mean	SD
Gestational Age (wks)	*N* = 498		*N* = 491	
	37.9	3.6	37.9	3.6
Birthweight (g)	*N* = 498		*N* = 493	
	2638.0	440.1	2593.5	494.8
Average # times massaged per day during 1st week of life	*N* = 491		*N* = 487	
	4.65	1.39	4.25	1.24

^1^Small for Gestational Age; ^2^ Adequate for Gestational Age

### Skin condition scores

**Fig. 2:**
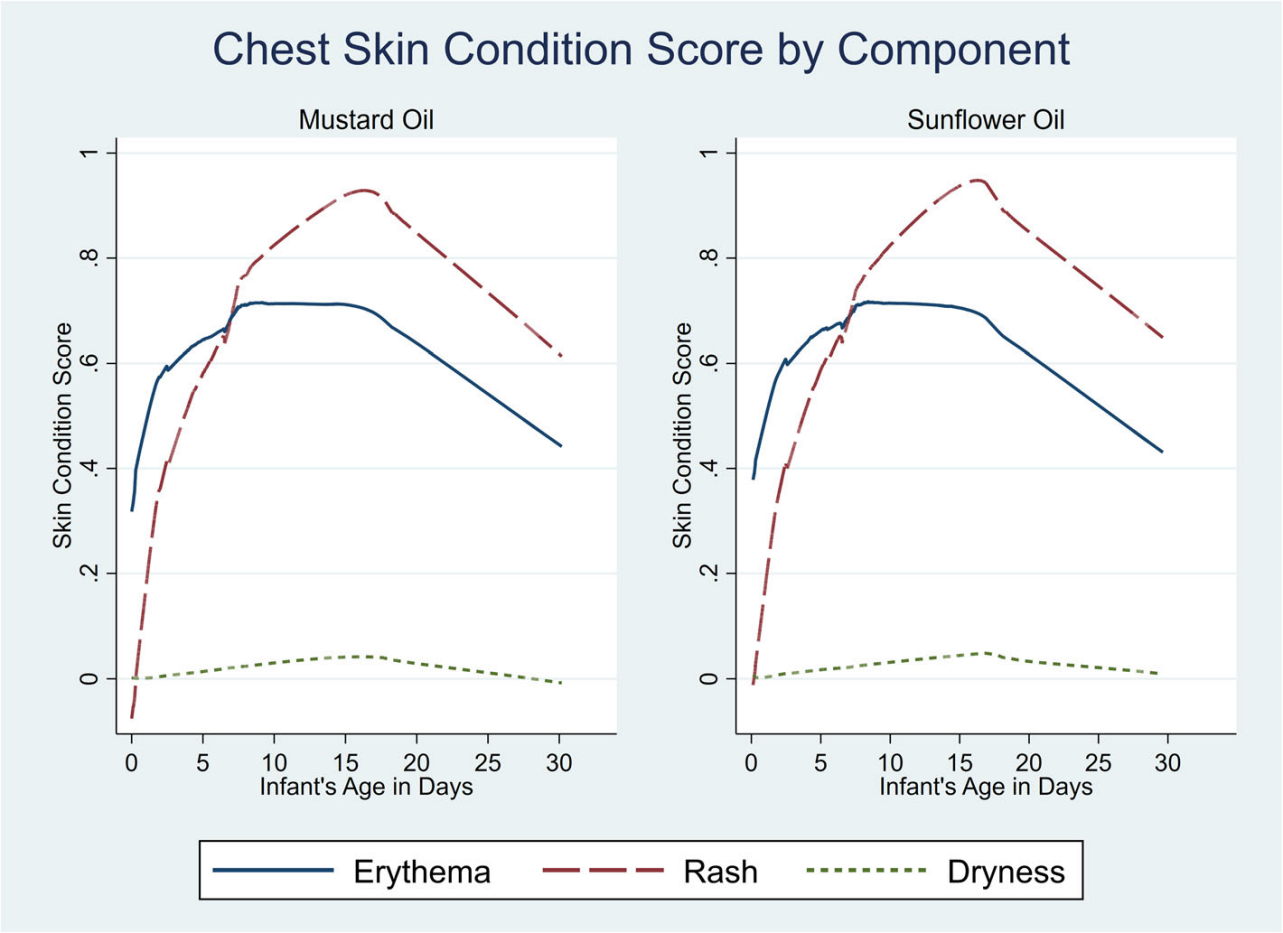
Lowess curves of change in chest skin condition throughout the neonatal period by oil group, Sarlahi, Nepal

At the initial visit, skin erythema, rash, and dryness scores did not differ between by group (Table 2) and followed a similar dynamic for both MO and SSO over the first 28 days). For both oils, scores increased (worsened) over days 1–14 followed by some improvement by day 28 (Fig. 2, chest). Rash was not present at birth, increased rapidly to day 14, then decreased. Erythema was seen at birth (day 1), increased to day 14 then decreased. Very little dryness was observed. Scores were comparable for chest, arm and leg sites and trends were similar by gestational age.

**Table 2:** Skin barrier integrity measures of neonates at five time points during the neonatal period, Sarlahi, Nepal

Skin Barrier Integrity Measure	Visit Number									
	1		3		7		14		28	
	MO	SSO	MO	SSO	MO	SSO	MO	SSO	MO	SSO
	Mean (SD)	Mean (SD)	Mean (SD)	Mean (SD)	Mean (SD)	Mean (SD)	Mean (SD)	Mean (SD)	Mean (SD)	Mean (SD)
Erythema Score, Chest	0.46 (0.61) *N* = 500	0.48 (0.62) *N* = 494	0.73 (0.63) *N* = 480	0.73 (0.62) *N* = 482	0.68 (0.59) *N* = 474	0.69 (0.62) *N* = 479	0.74 (0.63) *N* = 464	0.72 (0.61) *N* = 463	0.49 (0.54) *N* = 394	0.46 (0.55) *N* = 395
Erythema Score, Arm	0.55 (0.67) *N* = 500	0.53 (0.65) *N* = 494	0.77 (0.63) *N* = 480	0.77 (0.60) *N* = 482	0.80 (0.58) *N* = 474	0.84 (0.62) *N* = 479	0.90 (0.63) *N* = 464	0.90 (0.60) *N* = 463	0.61 (0.53) *N* = 394	0.57 (0.56) *N* = 395
Erythema Score, Leg	0.57 (0.68) *N* = 500	0.54 (0.67) *N* = 494	0.74 (0.65) *N* = 480	0.75 (0.65) *N* = 482	0.77 (0.61) *N* = 474	0.81 (0.64) *N* = 479	0.82 (0.62) *N* = 464	0.84 (0.60) *N* = 463	0.63 (0.59) *N* = 394	0.58 (0.61) *N* = 395
Rash Score, Chest	0.13 (0.39) *N* = 500	0.16 (0.43) *N* = 494	0.67 (0.76) *N* = 480	0.63 (0.75) *N* = 482	0.72 (0.77) *N* = 474	0.73 (0.80) *N* = 479	0.97 (0.79) *N* = 464	0.97 (0.80) *N* = 463	0.66 (0.70) *N* = 394	0.68 (0.74) *N* = 395
Rash Score, Arm	0.17 (0.47) *N* = 500	0.13 (0.40) *N* = 494	0.65 (0.76) *N* = 480	0.61 (0.76) *N* = 482	0.93 (0.84) *N* = 474	0.90 (0.88) *N* = 479	1.18 (0.87) *N* = 464	1.19 (0.87) *N* = 463	0.74 (0.72) *N* = 394	0.73 (0.74) *N* = 395
Rash Score, Leg	0.15 (0.44) *N* = 500	0.12 (0.37) *N* = 494	0.52 (0.70) *N* = 480	0.48 (0.69) *N* = 482	0.71 (0.78) *N* = 474	0.70 (0.77) *N* = 479	0.98 (0.78) *N* = 464	1.02 (0.82) *N* = 463	0.74 (0.74) *N* = 394	0.69 (0.74) *N* = 395
Dryness Score, Chest	0.002 (0.03) *N* = 500	0.004 (0.07) *N* = 494	0.010 (0.16) *N* = 480	0.016 (0.20) *N* = 482	0.017 (0.20) *N* = 474	0.029 (0.27) *N* = 479	0.050 (0.37) *N* = 464	0.038 (0.32) *N* = 463	0 *N* = 394	0.011 (0.16) *N* = 395
Dryness Score, Arm	0.004 (0.07) *N* = 500	0.018 (0.19) *N* = 494	0.067 (0.40) *N* = 480	0.049 (0.35) *N* = 482	0.16 (0.65) *N* = 474	0.15 (0.64) *N* = 479	0.15 (0.64) *N* = 464	0.27 (0.85) *N* = 463	0.020 (0.20) *N* = 394	0.029 (0.27) *N* = 395
Dryness Score, Leg	0.010 (0.15) *N* = 500	0.02 (0.26) *N* = 494	0.027 (0.23) *N* = 480	0.033 (0.29) *N* = 482	0.12 (0.56) *N* = 474	0.14 (0.63) *N* = 479	0.039 (0.31) *N* = 464	0.06 (0.40) *N* = 463	0.024 (0.26) *N* = 394	0.039 (0.33) *N* = 395
Skin pH	6.1 (0.53) *N* = 498	6.2 (0.53)^§^ *N* = 495	5.7 (0.53) *N* = 480	5.8 (0.52) *N* = 481	5.3 (0.58) *N* = 472	5.4 (0.56) *N* = 479	5.2 (0.57) *N* = 459	5.2 (0.56) *N* = 461	5.0 (0.61) *N* = 389	5.0 (0.60) *N* = 386
TEWL (g/m^2^/hr)	33.2 (23.5) *N* = 499	35.5 (23.8) *N* = 494	36.1 (24.7) *N* = 480	37.2 (24.6) *N* = 480	37.6 (24.5) *N* = 468	39.1 (25.1) *N* = 475	40.7 (24.3) *N* = 458	40.7 (24.1) *N* = 453	43.0 (24.5) *N* = 386	42.0 (23.2) *N* = 384
Protein concentration (μg/cm^2^)	16.6 (7.88) *N* = 497	17.9 (7.87)* *N* = 491	N/A	N/A	13.3 (6.52) *N* = 473	14.0 (6.74) *N* = 475	13.4 (6.96) *N* = 460	14.1 (6.78) *N* = 459	13.5 (5.89) *N* = 391	14.7 (6.03) ^§^ *N* = 388

*Abbreviations*: *SD* Standard deviation, *TEWL* Transepidermal water loss, *MO* Mustard oil, *SFO* Sunflower oil

*p < 0.05; § *p* < 0.01

### Skin pH, SC cohesion, and TEWL

At day 1, skin pH (6.1, 95% CI 6.05–6.14 [MO]; 6.2, 95% CI 6.15–6.28 [SSO]) and protein concentration (16.6 μg/cm^2^, 95% CI 15.9–17.3 [MO]; 17.8 μg/cm^2^, 95% CI 17.1–18.6 [SSO]) were significantly higher in the SSO group (Table 2); TEWL did not differ by group at baseline. The baseline differences in pH and protein concentration may be attributable to variances in environmental conditions on that day, time from birth, or dissimilarities in GA distribution. Treatment assignment could not practically include baseline values for stratification.

**Fig. 3:**
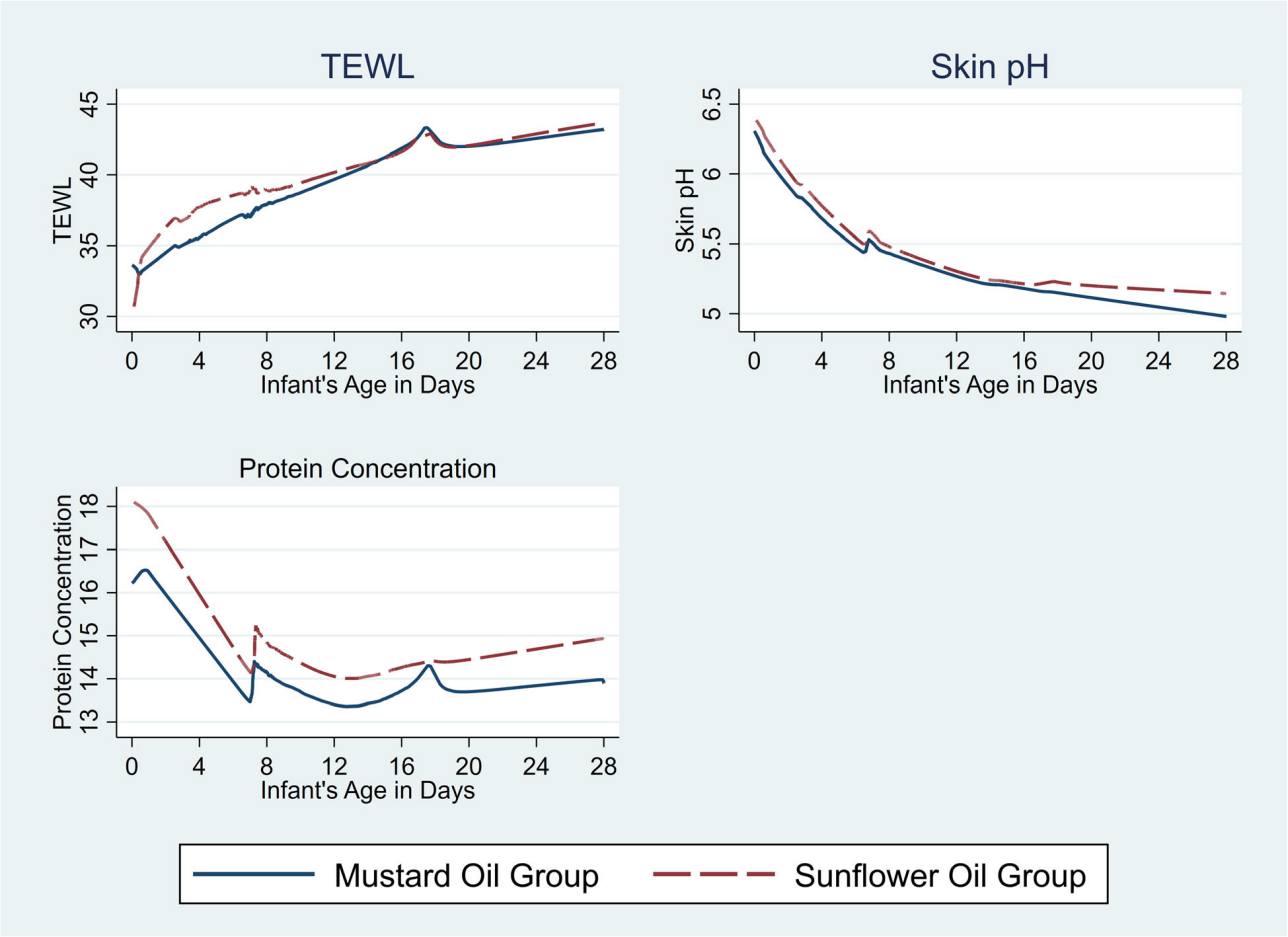
Lowess curves of the changes in TEWL, Skin pH, and SC protein concentration throughout the neonatal period, Sarlahi, Nepal

Mean pH decreased faster (i.e., maturation), 0.02 (95% CI 0.002–0.04) units per day more quickly, for SSO versus MO during the first week of life; there was not a significant difference in rate of decrease of pH in weeks 2–4 (Fig. 3). SC protein was higher for SSO at day 28 (i.e. versus day 14) and was significantly higher than MO [13.5 μg/cm^2^, 95% CI 12.8–14.1 (MO); 14.8 μg/cm^2^ 95% CI 14.1–15.4 (SSO)] (Table 2). TEWL increased significantly over time for both oils but did not differ between them (Fig. 3).

### Effect of oil treatment by gestational age group (Table 3)

Infants 34–36 weeks receiving SSO had a 0.14 (95% CI 0.024–0.25) higher average skin pH over 28 days than those with MO, but the rate of decrease in pH (i.e. maturation), which was higher in SSO infants overall, was not modified by gestational age. Infants 34–36 weeks and term infants receiving SSO had higher SC protein compared with MO; mean difference (i.e. increase in SSO group) was 1.27 μg/cm^2^ [95% CI: 0.33–2.21]) and 0.83 μg/cm^2^ (0.16–1.50) higher for SSO versus MO, respectively. Skin erythema, rash and TEWL did not differ by gestational age.

**Table 3:** Bivariate random-effects model comparing skin integrity measures throughout the entire study period by oil group, Sarlahi, Nepal

Skin Barrier Integrity Measure	Total		< 34 Weeks		34–36 Weeks		≥37 Weeks	
	*N* = 500 (MO)		*N* = 56 (MO)		*N* = 148 (MO)		*N* = 294 (MO)	
	*N* = 495 (SSO)		*N* = 56 (SSO)		*N* = 139 (SSO)		*N* = 296 (SSO)	
	β	95%CI	β	95%CI	β	95%CI	β	95%CI
TEWL (g/m^2^/hr)	1.11	−1.25,3.47	3.81	−3.23,10.85	−0.43	−4.56,3.69	1.09	−1.78,3.96
Skin pH	0.06	− 0.01,0.12	0.01	− 0.12,0.15	0.14^***^	0.024,0.25	0.03	−0.05,0.10
Protein concentration (μg/cm^2^)	0.97^*^	0.44,1.49	0.84	−0.79,2.46	1.27^**^	0.33,2.21	0.83^*^	0.16,1.50
Chest erythema score	−0.0003	−0.05,0.05	0.03	−0.11,0.18	− 0.008	−0.12,0.10	− 0.0001	−0.07,0.07
Chest rash score	0.003	−0.05,0.06	−0.15	− 0.30,0.007	0.003	− 0.10,0.11	0.03	− 0.04,0.10
Chest dryness score	0.004	−0.009,0.02	−0.03	− 0.07,0.008	0.01	− 0.008,0.03	0.006	− 0.01,0.02
Left arm erythema score	−0.0008	− 0.06,0.06	−0.0007	− 0.15,0.15	0.02	− 0.10,0.13	−0.007	− 0.08,0.06
Left arm rash score	−0.02	− 0.08,0.04	−0.15	− 0.32,0.02	0.01	− 0.11,0.13	−0.01	− 0.09,0.06
Left arm dryness score	0.02	−0.01,0.06	−0.03	− 0.12,0.07	0.01	− 0.04,0.06	0.04	− 0.01-0.09
Right leg erythema score	−0.001	− 0.06,0.06	0.04	− 0.12,0.21	−0.02	− 0.13,0.10	0.005	− 0.07,0.08
Right leg rash score	−0.02	− 0.07,0.04	−0.10	− 0.25,0.05	−0.01	− 0.11,0.09	−0.003	− 0.07,0.07
Right leg dryness score	0.02	−0.005,0.04	0.02	−0.006,0.04	0.01	−0.03,0.05	0.02	−0.01,0.05

^1^ Reference Group: Mustard Oil Group, *p < 0.001 **p < 0.01, ***p < 0.05

## Discussion

We compared the effects of emollient therapy with MO and SSO on neonatal skin barrier integrity in community settings of rural Nepal. Unlike prior work in this domain, we included both preterm and full-term infants, enrolled infants from birth and followed them prospectively through 28 days, and observed the impact of these emollients on skin barrier function and integrity at home in high relative humidity and temperature conditions. The emollient therapy involved consistent repetitive, daily, high frequency oil exposure with 4.2 (SSO) and 4.6 (MO) vigorous massages each day. This is in contrast to the hospital studies where SSO was applied 3 times daily for 14 days then twice daily until day 28 [9, 22].

Relative to traditional MO, use of SSO increased the rate of skin pH reduction during week one of life, suggesting a faster acid mantle development. The more rapid pH decrease for SSO may be protective for neonates in lower resource settings. An acidic environment is required for lipid metabolism, bilayer structure formation, desquamation, bacterial homeostasis, skin colonization, and inhibition of pathogenic bacteria [23–27]. The more rapid pH decrease with SSO, suggests that the pH decrease may favorably impact barrier normalization [28]. The influence of exogenous materials on adaptive mechanisms has been reported; treatment with peroxisome proliferator-activated receptor alpha (PPARα) activators increased the rate of skin pH lowering in neonatal animals [29]. Topical acidic treatments has been proposed for treating inflammation and normalizing SC structure [30].

For both groups, TEWL was higher (mean, 33.2–43.0 g/m^2^/hr) than in prior studies, with increasing water losses through the first three weeks. TEWL has largely been examined in controlled settings [31–34] with substantially lower humidity and temperature than our setting. Our higher, increasing TEWL may reflect eccrine gland maturation and/or water loss due to sweating. The increases may be due to skin compromise, i.e., rash, erythema. Determination of the effects of the oils alone, total daily oil exposure, the massage process or the combination of oil with massage would require a different experimental design than we used here. In preterm infants (*n* = 22) receiving sunflower oil massage at high daily frequency (every 3–4 h) from birth, TEWL increased significantly over 11 days but decreased when application was discontinued [35]. In our community, where daily massage with oil is nearly universal and the focus on comparing SSO with MO, we cannot determine whether TEWL values might have been lower or decreased upon discontinuing massage in a group of babies unexposed to either oil. TEWL decreased following three times weekly SSO application in full-term neonates (*n* = 24) over 5 weeks [36] but exposures were substantially lower than in our study.

Regardless of oil exposure, skin condition (especially rash) worsened through the first two weeks before decreasing in severity. However, we rarely observed any skin dryness or scaling, even in infants < 34 weeks, unlike the Bangladesh hospital-based emollient trial where skin dryness occurred in 59% of infants < 33 weeks gestational age [37]. Our contrasting finding may result from the high relative humidity, consistently between 80 and 95%, an optimal range for filaggrin proteolysis to natural moisturizing factor that can increase skin hydration [38]. In contrast with the Bangladesh trial, where preterm infants (GA: 31.2 wks) received topical SSO experienced improved skin condition versus no massage, we did not detect improvements, even among our youngest category. Our generally better skin condition, compared to the Bangladesh study where 59% of the subjects (premature infants only) had skin dryness or erythema [37] may account our lack of measurable improvements with SSO. In addition, as the worsening of skin condition during the first two weeks occurred in both oil groups and as we were unable to include a control group in this study for comparison, it is possible this could have been due to the use of oil or massage itself.

We hypothesized that the impact of SSO on skin barrier function might be modified by gestational age; specifically, we considered the possibility that any beneficial effects, such as those we observed overall on rate of skin pH decline, might be limited to or realized to a greater degree among preterm infants, given that younger preterm infants are most vulnerable to the consequences of an under-developed barrier, e.g., infection. Further, we previously reported that in this setting among babies exposed to traditional MO massage, skin pH was significantly higher for those < 34 weeks relative to older infants. There was no clear evidence, however, of consistently stronger effects of SSO by gestational age; in general, TEWL, skin pH, and skin condition in the SSO group relative to MO group did not differ by gestational age. The faster (i.e. unit decrease per day) decrease in pH among SSO infants was modest, and we did not find any evidence that this observed effect varied by gestational age. While the mean SC protein amounts were higher in the older preterm and full term SSO infants, suggesting possible increased desquamation among this group, the difference between the groups was small. For both oils and all gestational ages protein measures decreased over time, and the rate of decrease differed neither overall or within gestational age category. Further studies are needed to discern the specific mechanisms of barrier development in the youngest infants over time.

While a large sample size, oversampling of preterm infants, and repeated measures of skin integrity and function over the neonatal period were strengths, our study has a number of limitations. We did not have a group of babies not exposed to oil and/or massage, a group which might have provided further insights into potential advantages or disadvantages of oil massage. While such information may have provided additional insight into the mechanism of the oils, the parent study was examining if substituting sunflower seed oil for the traditionally-utilized mustard seed oil could lead to health benefits without introducing a major behavioral change to an important cultural practice [11]. Our population included premature infants. However, there were a small number (*n* = 14) of extremely premature infants (< 29 weeks) and preterms < 32 weeks GA (*n* = 54), thereby limiting the ability to discern emollient differences in the most underdeveloped skin. Strategies to facilitate barrier development in very premature babies are relevant in intensive care settings, and additional research is warranted. TEWL, one of our skin barrier integrity measures, is recommended for use in controlled conditions with low humidity [39, 40]. Any true benefit of SSO on TEWL might have been obscured in our high humidity conditions, through concomitant effects of sweating. Finally, given the constraints of the setting, we used reported date of last menstrual period, rather than ultrasound dating, resulting in some possible misclassification of gestational age [41].

## Conclusions

The application of topical emollients for preterm and full-term infants continues to be investigated worldwide. These data provide information about how different oils may facilitate skin barrier development and, thereby, protect infants in low resource, high humidity settings. These data and those from other studies further examining these mechanisms in a variety of settings and with different emollient regimens can help guide the development and/or section of more effective treatments. Emollients have been used to facilitate skin maturation in premature infants in hospital and rural settings, potentially lowering risk of life-threatening infections. Most recently, they have demonstrated potential to reduce the incidence of atopic dermatitis when administered right after birth [42, 43]. The present study adds knowledge by examining the mechanistic basis for the effect of emollient therapy on skin barrier function, and highlighting the role of environmental conditions such as relative humidity and temperature and cultural practices such as multiple daily emollient application with vigorous massage. The specific emollient composition likely impact the outcomes, suggesting the importance of identifying the characteristics of optimum topical treatments across conditions, such as atopic dermatitis where specific skin barrier anomalies are implicated in the disease process [44]. Overall, research on how topical emollients influence the infant skin barrier remains inconclusive, and future studies, e.g., on specific biomarkers of innate immunity, could clarify some inconsistencies. Investigations on blockage of eccrine glands by topical oils and influence skin microflora in these settings are warranted. Finally, the effects of the repetitive vigorous massage itself, regardless of oil might be used remains an under-studied aspect of emollient therapy research, particularly among younger premature infants for whom skin barrier maturation is incomplete.

## Supplementary information

**Additional file 1: Table S1.** Skin Condition Scale.

## Data Availability

The datasets used and analyzed for this study are available from the corresponding author upon request.

## Abbreviations

CI: Confidence Interval
GA: Gestational age
MO: Mustard seed oil
PPARα: Peroxisome proliferator activated receptor alpha
SC: Stratum corneum
SGA: Small for gestational age
SSO: Sunflower seed oil
TEWL: Trans-epidermal water loss
VDC: Village development committee

## References

[1] Darmstadt GL, Dinulos JG (2000). Neonatal skin care. Pediatr Clin N Am.

[2] Visscher MO, Adam R, Brink S, Odio M (2015). Newborn infant skin: physiology, development, and care. Clin Dermatol.

[3] Evans NJ, Rutter N (1986). Development of the epidermis in the newborn. Biol Neonate.

[4] Cartlidge P (2000). The epidermal barrier. Semin Neonatol.

[5] Lawn JE, Cousens S, Zupan J (2005). 4 million neonatal deaths: when? Where? Why?. Lancet.

[6] Belizan JM, McClure EM, Goudar SS, Pasha O, Esamai F, Patel A, Chomba E, Garces A, Wright LL, Koso-Thomas M (2012). Neonatal death in low- to middle-income countries: a global network study. Am J Perinatol.

[7] Darmstadt GL, Mao-Qiang M, Chi E, Saha SK, Ziboh VA, Black RE, Santosham M, Elias PM (2002). Impact of topical oils on the skin barrier: possible implications for neonatal health in developing countries. Acta Paediatr.

[8] Darmstadt GL, Saha SK, Ahmed AS, Ahmed S, Chowdhury MA, Law PA, Rosenberg RE, Black RE, Santosham M (2008). Effect of skin barrier therapy on neonatal mortality rates in preterm infants in Bangladesh: a randomized, controlled, clinical trial. Pediatrics.

[9] Darmstadt GL, Saha SK, Ahmed AS, Chowdhury MA, Law PA, Ahmed S, Alam MA, Black RE, Santosham M (2005). Effect of topical treatment with skin barrier-enhancing emollients on nosocomial infections in preterm infants in Bangladesh: a randomised controlled trial. Lancet.

[10] Ministry of Health N, ERA N, ICF (2017). Nepal Demographic and Health Survey 2016. Kathmandu, Nepal: Ministry of Health , Nepal.

[11] Mullany LC, Darmstadt GL, Khatry SK, Tielsch JM (2005). Traditional massage of newborns in Nepal: implications for trials of improved practice. J Trop Pediatr.

[12] Eichenfield LF, Hardaway CA (1999). Neonatal dermatology. Curr Opin Pediatr.

[13] Darmstadt GL (1998). The skin and nutritional disorders in the newborn. Eur J Pediatr Dermatol.

[14] Summers A, Visscher MO, Khatry SK, Sherchand JB, LeClerq SC, Katz J, Tielsch JM, Mullany LC (2018). Indicators of skin barrier integrity among newborns massaged with mustard oil in rural Nepal. J Perinatol.

[15] Lukacovic M, Dunlap RE, Visscher MO, Watson DD (1988). Forearm wash test to evaluate the clinical mildness of cleansing products. J Soc Cosmet Chem.

[16] Odio MR, O'Connor RJ, Sarbaugh F, Baldwin S (2000). Continuous topical administration of a petrolatum formulation by a novel disposable diaper. 2. Effect on skin condition. Dermatology.

[17] De Paepe K, Houben E, Adam R, Wiesemann F, Rogiers V (2005). Validation of the VapoMeter, a closed unventilated chamber system to assess transepidermal water loss vs. the open chamber Tewameter. Skin Res Technol.

[18] Voegeli R, Rawlings AV, Doppler S, Heiland J, Schreier T (2007). Profiling of serine protease activities in human stratum corneum and detection of a stratum corneum tryptase-like enzyme. Int J Cosmet Sci.

[19] Rothfusz LP (1990). The Heat Index “Equation” (or, More Than You Ever Wanted to Know About Heat Index). Fort Worth, TX:National Oceanic and Atmospheric Administration: National Weather Service, Office of Meteorology.

[20] Steadman RG (1979). The assessment of sultriness. Part I: a temperature-humidity index based on human physiology and clothing science. J Appl Meteorol.

[21] Katz J, Wu LA, Mullany LC, Coles CL, Lee AC, Kozuki N, Tielsch JM (2014). Prevalence of small-for-gestational-age and its mortality risk varies by choice of birth-weight-for-gestation reference population. PLoS One.

[22] Darmstadt GL, Badrawi N, Law PA, Ahmed S, Bashir M, Iskander I, Al Said D, El Kholy A, Husein MH, Alam A (2004). Topically applied sunflower seed oil prevents invasive bacterial infections in preterm infants in Egypt: a randomized, controlled clinical trial. Pediatr Infect Dis J.

[23] Aly R, Shirley C, Cunico B, Maibach HI (1978). Effect of prolonged occlusion on the microbial flora, pH, carbon dioxide and transepidermal water loss on human skin. J Invest Dermatol.

[24] Fluhr JW, Kao J, Jain M, Ahn SK, Feingold KR, Elias PM (2001). Generation of free fatty acids from phospholipids regulates stratum corneum acidification and integrity. J Invest Dermatol.

[25] Puhvel SM, Reisner RM, Amirian DA (1975). Quantification of bacteria in isolated pilosebaceous follicles in normal skin. J Invest Dermatol.

[26] Rippke F, Schreiner V, Schwanitz HJ (2002). The acidic milieu of the horny layer: new findings on the physiology and pathophysiology of skin pH. Am J Clin Dermatol.

[27] Schmid-Wendtner MH, Korting HC (2006). The pH of the skin surface and its impact on the barrier function. Skin Pharmacol Physiol.

[28] Elias PM (2017). The how, why and clinical importance of stratum corneum acidification. Exp Dermatol.

[29] Fluhr JW, Man MQ, Hachem JP, Crumrine D, Mauro TM, Elias PM, Feingold KR (2009). Topical peroxisome proliferator activated receptor activators accelerate postnatal stratum corneum acidification. J Invest Dermatol.

[30] Hachem JP, Roelandt T, Schurer N, Pu X, Fluhr J, Giddelo C, Man MQ, Crumrine D, Roseeuw D, Feingold KR (2010). Acute acidification of stratum corneum membrane domains using polyhydroxyl acids improves lipid processing and inhibits degradation of corneodesmosomes. J Invest Dermatol.

[31] Garcia Bartels N, Mleczko A, Schink T, Proquitte H, Wauer RR, Blume-Peytavi U (2009). Influence of bathing or washing on skin barrier function in newborns during the first four weeks of life. Skin Pharmacol Physiol.

[32] Garcia Bartels N, Scheufele R, Prosch F, Schink T, Proquitte H, Wauer RR, Blume-Peytavi U (2010). Effect of standardized skin care regimens on neonatal skin barrier function in different body areas. Pediatr Dermatol.

[33] Minami-Hori M, Honma M, Fujii M, Nomura W, Kanno K, Hayashi T, Nakamura E, Nagaya K, Miyauchi Y, Fujimura T (2014). Developmental alterations of physical properties and components of neonatal-infantile stratum corneum of upper thighs and diaper-covered buttocks during the 1st year of life. J Dermatol Sci.

[34] Visscher M, Odio M, Taylor T, White T, Sargent S, Sluder L, Smith L, Flower T, Mason B, Rider M (2009). Skin care in the NICU patient: effects of wipes versus cloth and water on stratum corneum integrity. Neonatology.

[35] Kanti V, Grande C, Stroux A, Buhrer C, Blume-Peytavi U, Garcia Bartels N (2014). Influence of sunflower seed oil on the skin barrier function of preterm infants: a randomized controlled trial. Dermatology.

[36] Kanti Varvara, Günther Malise, Stroux Andrea, Sawatzky Sabine, Henrich Wolfgang, Abou-Dakn Michael, Blume-Peytavi Ulrike, Garcia Bartels Natalie (2017). Influence of sunflower seed oil or baby lotion on the skin barrier function of newborns: A pilot study. Journal of Cosmetic Dermatology.

[37] Darmstadt GL, Ahmed S, Ahmed AS, Saha SK (2014). Mechanism for prevention of infection in preterm neonates by topical emollients: a randomized, controlled clinical trial. Pediatr Infect Dis J.

[38] Scott IR, Harding CR (1986). Filaggrin breakdown to water binding compounds during development of the rat stratum corneum is controlled by the water activity of the environment. Dev Biol.

[39] du Plessis J, Stefaniak A, Eloff F, John S, Agner T, Chou TC, Nixon R, Steiner M, Franken A, Kudla I (2013). International guidelines for the in vivo assessment of skin properties in non-clinical settings: part 2. Transepidermal water loss and skin hydration. Skin Res Technol.

[40] Pinnagoda J, Tupker RA, Agner T, Serup J (1990). Guidelines for transepidermal water loss (TEWL) measurement. A report from the standardization Group of the European Society of contact dermatitis. Contact Dermatitis.

[41] Rosenberg RE, Ahmed AS, Ahmed S, Saha SK, Chowdhury MA, Black RE, Santosham M, Darmstadt GL (2009). Determining gestational age in a low-resource setting: validity of last menstrual period. J Health Popul Nutr.

[42] Horimukai K, Morita K, Narita M, Kondo M, Kitazawa H, Nozaki M, Shigematsu Y, Yoshida K, Niizeki H, Motomura K (2014). Application of moisturizer to neonates prevents development of atopic dermatitis. J Allergy Clin Immunol.

[43] Simpson EL, Chalmers JR, Hanifin JM, Thomas KS, Cork MJ, McLean WH, Brown SJ, Chen Z, Chen Y, Williams HC (2014). Emollient enhancement of the skin barrier from birth offers effective atopic dermatitis prevention. J Allergy Clin Immunol.

[44] Glatz M, Jo JH, Kennedy EA, Polley EC, Segre JA, Simpson EL, Kong HH (2018). Emollient use alters skin barrier and microbes in infants at risk for developing atopic dermatitis. PLoS One.

