# Supplementary material for: Impact of sunflower seed oil versus mustard seed oil on skin barrier function in newborns: a community-based, cluster-randomized trial
Source: BMC Pediatr. 2019 Dec 23;19:512. doi: 10.1186/s12887-019-1871-2 (PMC6927111; doi:10.1186/s12887-019-1871-2)
Supplement: Supplementary file 1 — Additional file 1: Table S1. Skin Condition Scale. [file 12887_2019_1871_MOESM1_ESM.docx]

Table S1: Skin Condition Scale

|  | **Severity** | **Area** |
| --- | --- | --- |
| **Erythema Scale** | | |
| 0 | None | None |
| 1 | Faint or definite pink | <2% |
| 2 | Definite red | 2-10% |
| 3 | Very intense redness | 10-50% |
| 4 | NA | >50% |
| **Rash Scale** | | |
| 0 | None | None |
| 1 | Papules | One |
| 2 | Pustules | 2-5 |
| 3 | Papules and pustules | <10% |
| 4 | Clear fluid-filled vesicles | 10-50% |
| 5 | NA | >50% |
| 6 | NA | Numerous and continuous/joining |
| **Dryness Scale** | | |
| 0 | None | None |
| 1 | Slight powderiness | <10% |
| 2 | Early cracking | 10-50% |
| 3 | Moderate cracking & scales | >50% |
| 4 | High cracking & lifting scales | NA |
| 5 | Bleeding cracks | NA |
